# Excessive tibial valgus was not associated with worse 5-year clinical outcomes after medial opening-wedge high tibial osteotomy for advanced knee osteoarthritis: a retrospective cohort study

**DOI:** 10.3389/fsurg.2026.1946486

**Published:** 2026-09-21

**Authors:** Yisan Wang, Guorui Cao, Xiao Wang, Peizhao Wang, Xiaotao Shi, Mingyang Zhang, Honglue Tan

**Affiliations:** 1 Department of Orthopedics, Henan University of Chinese Medicine Henan Provincial Hospital of TCM, Zhengzhou, China; 2 Department of knee surgery, Luoyang Orthopedic Traumatological Hospital, Luoyang, China

**Keywords:** clinical outcomes, Kellgren-Lawrence grade IV, knee osteoarthritis, medial opening-wedge high tibial osteotomy, medial proximal tibial angle, tibial valgus

## Abstract

**Purpose:**

To evaluate whether excessive tibial valgus, defined as a medial proximal tibial angle (MPTA) > 95° at the final follow-up, was associated with worse 5-year clinical outcomes after medial opening-wedge high tibial osteotomy (MOWHTO) in patients with Kellgren-Lawrence (K-L) grade IV medial compartment knee osteoarthritis.

**Methods:**

This retrospective cohort study included 165 knees with K-L grade IV medial compartment knee osteoarthritis that underwent MOWHTO. Knees were classified according to the MPTA measured on weight-bearing full-length radiographs at the final follow-up as MPTA >95° (*n* = 81) or MPTA ≤95° (*n* = 84). The primary outcome was the Western Ontario and McMaster Universities Osteoarthritis Index (WOMAC) score at the final follow-up. Secondary outcomes included the Lysholm score, visual analogue scale (VAS) for pain, knee range of motion (ROM), and radiographic parameters. Between-group changes were compared, and clinical outcomes were further evaluated using baseline-adjusted analysis of covariance.

**Results:**

The mean follow-up duration was 64.49 ± 3.60 months. WOMAC, Lysholm, VAS, and ROM improved significantly from baseline to the final follow-up in the overall cohort (all *P* < 0.001). At the final follow-up, WOMAC, Lysholm, VAS, and ROM did not differ significantly between the MPTA >95° and MPTA ≤95° groups. Although changes in MPTA and hip–knee–ankle angle were greater in the MPTA >95° group, no significant between-group differences were observed in changes in WOMAC, Lysholm, VAS, or ROM after Holm adjustment. In baseline-adjusted analyses, an MPTA >95° was not significantly associated with WOMAC (adjusted mean difference, −0.16; 95% CI, −5.42 to 5.11; *P* = 0.953), Lysholm (0.24; 95% CI, −3.22 to 3.69; *P* = 0.893), VAS (−0.07; 95% CI, −0.39 to 0.24; *P* = 0.645), or ROM (−1.52°; 95% CI, −4.44 to 1.39; *P* = 0.306).

**Conclusion:**

In patients with K-L grade IV medial compartment knee osteoarthritis treated with MOWHTO, an MPTA >95° at the final follow-up was not significantly associated with worse 5-year clinical outcomes. These findings suggest that the 95° MPTA threshold should not be interpreted in isolation as a determinant of clinical outcome; however, they do not define an optimal correction target or support deliberate overcorrection beyond 95°.

## 1 Introduction

Medial opening-wedge high tibial osteotomy (MOWHTO) is a joint-preserving treatment for symptomatic medial compartment knee osteoarthritis (OA) associated with varus malalignment. By correcting the proximal tibial alignment, MOWHTO redistributes load away from the diseased medial compartment while preserving the native knee joint, making it an important option for selected patients with high functional demands (1). Although HTO has traditionally been used more often in patients with less advanced OA, its role in advanced disease has received increasing attention. A 2024 systematic review of radiographically advanced medial compartment OA reported favorable mid- to long-term survivorship and clinically meaningful improvement in patient-reported outcomes after HTO (2). Long-term data have also shown satisfactory clinical and radiographic outcomes after MOWHTO in carefully selected younger patients with advanced medial compartment OA, supporting joint-preserving surgery as a reasonable option in this population (3).

The optimal magnitude of correction after MOWHTO, however, remains controversial. The medial proximal tibial angle (MPTA) is commonly used to characterize the coronal orientation of the proximal tibia, and a postoperative MPTA of approximately 95° has frequently been used to identify excessive tibial valgus, partly because of concerns regarding increased joint-line obliquity (JLO) (4). A systematic review found that the available evidence was insufficient to establish a consistent association between postoperative JLO and clinical outcomes or surgical survival after HTO. Excessive postoperative JLO has been associated with inferior radiographic and clinical outcomes after MOWHTO (5). In contrast, a propensity score-matched study found that an increased postoperative MPTA did not adversely affect overall mid-term clinical outcomes, although lateral compartment pain was more frequent in knees with greater joint-line obliquity (6). Similarly, a study with an average 10-year follow-up reported that an MPTA ≥95° was not associated with inferior WOMAC or Knee Society scores after opening-wedge HTO (7).

More recent evidence further suggests that an MPTA ≥95° should not automatically be interpreted as excessive postoperative joint-line inclination. Hizume et al. found that although postoperative MPTA was positively associated with knee JLO, more than half of knees with an MPTA ≥95° maintained a joint-line obliquity of <5° (8). These findings highlight that the clinical significance of the 95° threshold remains uncertain and may not be adequately explained by radiographic alignment alone. Importantly, studies evaluating HTO in advanced medial compartment OA have commonly included heterogeneous radiographic populations or combined Kellgren–Lawrence (K-L) grade III and IV knees, and evidence focused specifically on K-L grade IV disease remains limited (2, 3). Therefore, the purpose of this retrospective cohort study was to evaluate whether an MPTA >95° at the final follow-up after MOWHTO was associated with worse 5-year clinical outcomes in patients with K-L grade IV medial compartment knee OA. We hypothesized that an MPTA >95° at the final follow-up would not be associated with worse 5-year clinical outcomes.

## 2 Materials and methods

### 2.1 Study design and patients

This was a single-center retrospective cohort study of patients with medial compartment knee osteoarthritis (OA) and varus malalignment who underwent medial opening-wedge high tibial osteotomy (MOWHTO) at our institution between 2016 and 2020. The study protocol was approved by the Medical Ethics Committee of our institution (Approval No. 2025KYKT0031-02). All participants provided written, informed consent.

Patients were included if they met the following criteria: (1) symptomatic medial compartment knee OA with varus malalignment and an indication for MOWHTO; (2) K-L grade IV OA of the medial compartment on preoperative weight-bearing radiographs; (3) primary MOWHTO performed at our institution; (4) available preoperative and final follow-up weight-bearing full-length lower-limb radiographs; (5) complete clinical outcome data at the required assessment time points; (6) Patients whose surgery was performed by the same surgical team.

Patients were excluded if they had (1) clinically relevant cruciate ligament injury or ligamentous instability; (2) advanced lateral compartment or patellofemoral OA; (3) severe osteoporosis; (4) previous surgery on the affected knee; (5) inflammatory arthritis, including rheumatoid arthritis; (6) secondary OA, including post-traumatic or post-infectious OA; (7) gait disorders secondary to hip or ankle pathology; (8) neuromuscular disorders affecting lower-limb function; (9) an active systemic infection or open wound at the surgical site; (10) incomplete radiographic or clinical data; (11) pregnancy; and (12) follow-up shorter than 5 years.

K-L grading was independently performed by two orthopedic surgeons using preoperative weight-bearing anteroposterior knee radiographs. Both observers were blinded to the final clinical outcomes. Interobserver reliability was assessed using the weighted Cohen *κ* coefficient. Disagreements were resolved by consensus, and eligibility was determined according to the consensus K-L grade. All knees included in the final cohort were confirmed as K-L grade IV.

A total of 180 knees were retrospectively screened. Fifteen knees without an available follow-up of at least 5 years were excluded, leaving 165 knees from 165 patients, including 0 bilateral cases, for the final analysis. For the present analysis, knees were classified according to the MPTA measured on weight-bearing full-length lower-limb radiographs at the final follow-up. Knees with an MPTA >95° were assigned to the excessive tibial valgus group (*n* = 81), whereas those with an MPTA ≤95° were assigned to the comparison group (*n* = 84).

### 2.2 Surgical procedure and postoperative management

All procedures were performed by orthopedic surgeons specializing in knee surgery within the same surgical team, using a standardized surgical technique. Preoperative weight-bearing radiographs and magnetic resonance imaging were routinely evaluated to confirm the surgical indication and assess the medial, lateral, and patellofemoral compartments. All procedures were performed with the patient in the supine position under anesthesia. Diagnostic arthroscopy was first performed through standard anteromedial and anterolateral portals to evaluate the articular cartilage and menisci. Unstable cartilage fragments and hypertrophic synovial tissue were debrided when necessary. Meniscal tears were treated with repair or partial meniscectomy according to tear pattern and tissue quality.

MOWHTO was subsequently performed through a medial proximal tibial approach. After exposure of the medial proximal tibia, two guidewires were inserted toward the proximal tibiofibular region under fluoroscopic guidance. The osteotomy was performed while preserving approximately 10 mm of the lateral cortical hinge. The osteotomy gap was gradually opened under fluoroscopic control until the planned coronal alignment was achieved. Lower-limb alignment was checked intraoperatively using a radiopaque alignment rod extending from the femoral head to the ankle center. The osteotomy was then stabilized with a medial locking plate and screws. Bone graft material was used when the osteotomy gap exceeded 15 mm. Final alignment and implant position were confirmed fluoroscopically before wound closure.

Postoperative management followed a standardized rehabilitation protocol. Ankle-pump and quadriceps-strengthening exercises were initiated on the first postoperative day. Partial weight-bearing with crutches and active knee range-of-motion exercises were started from the second postoperative day as tolerated. Sutures were removed approximately 2 weeks after surgery. Full weight-bearing was generally permitted 4-6 weeks postoperatively according to clinical and radiographic healing, and patients gradually returned to unrestricted daily activities thereafter.

### 2.3 Radiographic and clinical assessments

Radiographic evaluations were performed preoperatively and at the final follow-up. Weight-bearing full-length anteroposterior radiographs of the lower limbs and weight-bearing anteroposterior and lateral radiographs of the knee were evaluated. The radiographic parameters included the MPTA, hip–knee–ankle angle (HKA), joint line convergence angle (JLCA), and posterior tibial slope angle (PTSA).

The MPTA was defined as the medial angle between the tibial mechanical axis and the tangent to the tibial plateau (9). The HKA angle was defined by the mechanical axes of the femur and tibia, with 180° representing neutral mechanical alignment, values <180° indicating varus alignment, and values >180° indicating valgus alignment. The JLCA was measured as the angle between the tangential line connecting the distal femoral condyles and the tangential line of the tibial plateau (10). The PTSA was measured on the lateral knee radiograph as the angle between the tibial plateau line and a line perpendicular to the tibial anatomical axis (11).

Radiographic measurements were performed independently by two orthopedic surgeons who were blinded to the clinical outcome data. Interobserver reliability for continuous radiographic measurements was assessed using intraclass correlation coefficients (ICCs). To assess intraobserver reliability, a randomly selected subset of knees was remeasured by both observers after an interval of at least 2 weeks, without access to the initial measurements. ICCs were calculated using an absolute-agreement model with 95% confidence intervals (12).

Clinical outcomes were assessed preoperatively and at the final follow-up using the Western Ontario and McMaster Universities Osteoarthritis Index (WOMAC), Lysholm Knee Score, visual analogue scale (VAS) for pain, and knee range of motion (ROM). The WOMAC was scored on a 0-240 scale, with higher scores indicating greater pain, stiffness, and functional impairment (13). The VAS ranged from 0 to 10, with higher scores indicating greater pain. Knee ROM was recorded in degrees.

### 2.4 Primary and secondary outcomes

The primary clinical outcome for the present analysis was the WOMAC score at the final follow-up. The principal comparison evaluated whether the final-follow-up WOMAC score differed between knees with a final-follow-up MPTA >95° and those with an MPTA ≤95°, with adjustment for the corresponding preoperative WOMAC score.

Secondary clinical outcomes included the Lysholm score, VAS pain score, and knee ROM at the final follow-up. Secondary radiographic outcomes included MPTA, HKA, JLCA, and PTSA. Changes in radiographic and clinical outcomes from baseline to final follow-up were calculated as the final-follow-up value minus the corresponding preoperative value.

Postoperative safety outcomes included symptomatic thrombosis, wound complications, and conversion to total knee arthroplasty during follow-up. No radiographic or clinical outcome data were missing among the 165 knees included in the final analysis.

### 2.5 Statistical analysis

Statistical analyses were performed using SPSS version 27.0 (IBM Corp., Armonk, NY, USA) and Python. Continuous variables are presented as mean ± standard deviation (SD), and categorical variables as number and percentage. Standardized mean differences (SMDs) were used to describe between-group differences in baseline characteristics. Preoperative and final-follow-up measurements within the same knees were compared using paired-samples t tests or Wilcoxon signed-rank tests, as appropriate. Between-group comparisons were performed using Welch t tests or Mann–Whitney U tests for continuous variables and chi-square or Fisher exact tests for categorical variables. Between-group comparisons of change scores were performed using Welch t tests, with *P* values adjusted for the eight comparisons using the Holm method. The association between final-follow-up MPTA group and clinical outcomes was further evaluated using analysis of covariance (ANCOVA), adjusting for the corresponding preoperative outcome. HC3 robust standard errors were used. All tests were two-sided, and *P* < 0.05 was considered statistically significant.

## 3 Results

Of the 180 knees screened, 15 were excluded because follow-up of at least 5 years was unavailable, leaving 165 knees for the final analysis, with 81 knees in the final-follow-up MPTA >95° group and 84 knees in the MPTA ≤95° group. The mean follow-up duration was 64.49 ± 3.60 months (range, 60–78 months). Interobserver agreement for K-L grading was substantial (weighted *κ*=0.71, 95% CI 0.63–0.82), and all included knees were confirmed as K-L grade IV after consensus assessment. Baseline demographic and perioperative characteristics are summarized in Table 1.

**Table 1:** Baseline demographic and perioperative characteristics according to final-follow-up MPTA group.

Characteristic	MPTA >95° (*n* = 81)	MPTA ≤95° (*n* = 84)	SMD	*P* value
Age, years	54.86 ± 4.72	54.77 ± 7.05	0.015	0.815
BMI, kg/m^2^	24.70 ± 2.92	24.25 ± 3.13	0.147	0.574
K-L grade IV, *n* (%)	81 (100)	84 (100)	——	——
Sex, *n* (%)			0.252	0.105
Male	42 (51.9)	33 (39.3)		
Female	39 (48.1)	51 (60.7)		
Operated side, *n* (%)			0.334	0.061
Left	49 (60.5)	37 (44.0)		
Right	32 (39.5)	47 (56.0)		
Operative time, min	121.79 ± 21.70	121.49 ± 33.85	0.011	0.703
Postoperative hospital stay, days	9.62 ± 3.22	9.61 ± 3.45	0.003	0.967
Follow-up Duration (months)	64.33 ± 3.47	64.64 ± 3.74	0.086	0.595

Continuous variables are presented as mean ± SD and were compared using the Mann–Whitney U test because of non-normal distributions. Categorical variables were compared using the chi-square test. SMD and *P* value were not calculated for K-L grade because all included knees were K-L grade IV. SMD, standardized mean difference; MPTA, medial proximal tibial angle.

Interobserver and intraobserver reliability for the radiographic measurements was good to excellent, with ICCs ranging from 0.88 to 0.94 and from 0.90 to 0.96, respectively. In the entire cohort, MPTA increased from 84.64 ± 3.53° preoperatively to 94.07 ± 2.69° at final follow-up, and HKA increased from 172.62 ± 4.21° to 180.69 ± 3.61° (both *P* < 0.001). PTSA also increased significantly (8.08 ± 2.92° vs. 8.62 ± 3.01°, *P* < 0.001). In contrast, JLCA did not change significantly (3.27 ± 1.77° vs. 3.21 ± 2.05°, *P* = 0.496). WOMAC, Lysholm, VAS, and ROM all improved significantly at final follow-up (all *P* < 0.001) (Table 2).

**Table 2:** Comparison of preoperative and final follow-up radiographic and clinical outcomes in the entire cohort (*n* = 165).

Outcome	Preoperative	Final follow-up	Change (post−pre)	*P* value
MPTA,°	84.64 ± 3.53	94.07 ± 2.69	9.43 ± 3.83	<0.001
JLCA,°	3.27 ± 1.77	3.21 ± 2.05	−0.06 ± 2.49	0.496
HKA,°	172.62 ± 4.21	180.69 ± 3.61	8.07 ± 4.62	<0.001
PTSA,°	8.08 ± 2.92	8.62 ± 3.01	0.54 ± 1.61	<0.001
WOMAC, points	129.19 ± 10.91	63.07 ± 16.97	−66.13 ± 20.21	<0.001
Lysholm, points	23.10 ± 4.72	75.85 ± 11.12	52.75 ± 12.10	<0.001
VAS, points	7.50 ± 0.97	1.53 ± 1.02	−5.96 ± 1.42	<0.001
ROM,°	98.85 ± 6.31	125.45 ± 9.46	26.61 ± 10.95	<0.001

Values are presented as mean ± SD. Change was calculated as the final-follow-up value minus the corresponding preoperative value. The preoperative-to-final-follow-up comparison of JLCA was performed using the Wilcoxon signed-rank test; all other outcomes were compared using paired-samples t tests. MPTA, medial proximal tibial angle; JLCA, joint line convergence angle; HKA, hip-knee-ankle angle; PTSA, posterior tibial slope angle; WOMAC, Western Ontario and McMaster Universities Osteoarthritis Index; VAS, visual analogue scale; ROM, range of motion.

At baseline, the MPTA >95° group had a greater preoperative MPTA than the MPTA ≤95° group (85.35 ± 3.93° vs. 83.95 ± 2.95°, *P* = 0.001). At final follow-up, MPTA and HKA were significantly greater in the MPTA >95° group, whereas JLCA, PTSA, WOMAC, Lysholm, VAS, and ROM did not differ significantly between groups (Table 3).

**Table 3:** Preoperative and final-follow-up outcomes according to final-follow-up MPTA group.

Outcome	Preoperative MPTA >95° (*n* = 81)	Preoperative MPTA ≤95° (*n* = 84)	Preoperative *P* value	Final follow-up MPTA >95° (*n* = 81)	Final follow-up MPTA ≤95° (*n* = 84)	Final follow-up *P* value
MPTA,°	85.35 ± 3.93	83.95 ± 2.95	0.001	96.45 ± 0.87	91.77 ± 1.64	<0.001
JLCA,°	3.42 ± 1.83	3.13 ± 1.71	0.322	2.88 ± 1.75	3.53 ± 2.27	0.097
HKA,°	173.02 ± 3.86	172.24 ± 4.51	0.093	182.12 ± 2.81	179.31 ± 3.77	<0.001
PTSA,°	7.75 ± 2.88	8.39 ± 2.95	0.161	8.51 ± 3.04	8.73 ± 2.98	0.643
WOMAC, points	129.06 ± 10.91	129.32 ± 10.97	0.879	62.99 ± 16.58	63.14 ± 17.45	0.876
Lysholm, points	23.04 ± 4.94	23.17 ± 4.51	0.861	75.98 ± 11.07	75.74 ± 11.24	0.913
VAS, points	7.60 ± 0.89	7.39 ± 1.04	0.136	1.49 ± 1.01	1.57 ± 1.02	0.693
ROM,°	98.95 ± 6.46	98.75 ± 6.20	0.837	124.69 ± 10.29	126.19 ± 8.59	0.304

Values are presented as mean ± SD. Welch independent-samples t tests were used for preoperative PTSA and WOMAC and for final-follow-up PTSA. Mann–Whitney U tests were used for all other between-group comparisons. MPTA, medial proximal tibial angle; JLCA, joint line convergence angle; HKA, hip-knee-ankle angle; PTSA, posterior tibial slope angle; WOMAC, Western Ontario and McMaster Universities Osteoarthritis Index; VAS, visual analogue scale; ROM, range of motion.

The between-group difference in *Δ*JLCA was significant before multiplicity adjustment (unadjusted *P* = 0.013) but was no longer significant after Holm correction (Holm-adjusted *P* = 0.079). After Holm adjustment, significant between-group differences remained for *Δ*MPTA (3.28°, 95% CI 2.21–4.36; *P* < 0.001) and *Δ*HKA (2.03°, 95% CI 0.64–3.41; *P* = 0.032), whereas no significant differences were observed for the remaining change scores (Table 4).

**Table 4:** Changes From baseline to final follow-up according to final-follow-up MPTA group.

Outcome	MPTA >95° *Δ* mean ± SD (*n* = 81)	MPTA ≤95° *Δ* mean ± SD (*n* = 84)	Between-group mean difference (95% CI)	Unadjusted *P*	Holm-adjusted *P* value
ΔMPTA,°	11.10 ± 3.89	7.82 ± 3.01	3.28 (2.21 to 4.36)	<0.001	<0.001
ΔJLCA,°	−0.54 ± 2.21	0.41 ± 2.65	−0.95 (−1.70 to −0.20)	0.013	0.079
ΔHKA,°	9.10 ± 4.51	7.07 ± 4.52	2.03 (0.64 to 3.41)	0.005	0.032
ΔPTSA,°	0.76 ± 1.33	0.34 ± 1.84	0.42 (−0.07 to 0.91)	0.093	0.464
ΔWOMAC, points	−66.07 ± 21.09	−66.18 ± 19.45	0.10 (−6.14 to 6.35)	0.974	1.000
ΔLysholm, points	52.94 ± 12.68	52.57 ± 11.58	0.37 (−3.37 to 4.10)	0.847	1.000
ΔVAS, points	−6.11 ± 1.36	−5.82 ± 1.47	−0.29 (−0.73 to 0.15)	0.191	0.765
ΔROM,°	25.74 ± 11.89	27.44 ± 9.95	−1.70 (−5.08 to 1.68)	0.322	0.965

Δ was defined as final follow-up minus the corresponding preoperative value. Between-group mean differences, 95% CIs, and unadjusted *P* values were estimated using Welch independent-samples t tests. Because eight change-score outcomes were examined simultaneously, the unadjusted *P* values were further adjusted using the Holm method to control the family-wise error rate. The reported 95% CIs are unadjusted for multiplicity.

In baseline-adjusted ANCOVA models, final-follow-up MPTA >95° was not significantly associated with WOMAC, Lysholm, VAS, or ROM outcomes. The adjusted between-group differences were −0.16 points for WOMAC (95% CI −5.42 to 5.11; *P* = 0.953), 0.24 points for Lysholm (95% CI −3.22 to 3.69; *P* = 0.893), −0.07 points for VAS (95% CI −0.39 to 0.24; *P* = 0.645), and −1.52° for ROM (95% CI −4.44 to 1.39; *P* = 0.306) (Table 5).

**Table 5:** Association between final-follow-up MPTA >95° and final-follow-up clinical outcomes.

Outcome	Unadjusted mean difference (95% CI)	*P* value	Baseline-adjusted mean difference (95% CI)	*P* value
WOMAC, points	−0.16 (−5.39 to 5.08)	0.953	−0.16 (−5.42 to 5.11)	0.953
Lysholm, points	0.24 (−3.19 to 3.67)	0.892	0.24 (−3.22 to 3.69)	0.893
VAS, points	−0.08 (−0.39 to 0.24)	0.625	−0.07 (−0.39 to 0.24)	0.645
ROM,°	−1.50 (−4.42 to 1.42)	0.312	−1.52 (−4.44 to 1.39)	0.306

Differences are MPTA >95° minus MPTA ≤95°. Unadjusted mean differences, 95% CIs, and *P* values were estimated using Welch independent-samples t tests. Baseline-adjusted estimates were obtained from ANCOVA models including the corresponding preoperative outcome; heteroscedasticity-consistent (HC3) standard errors were used. Therefore, unadjusted *P* values in this table may differ from the Mann–Whitney U test *P* values reported for the corresponding raw between-group comparisons in Table 3. CI, confidence interval.

Postoperative thrombosis occurred in 2 knees (2.5%) in the MPTA >95° group and 3 knees (3.6%) in the MPTA ≤95° group (*P* = 1.000). Wound complications occurred in 6 (7.4%) and 7 (8.3%) knees, respectively (*P* = 0.827). Among the 165 knees included in the final analysis, all of which had at least 5 years of follow-up, no knee underwent conversion to total knee arthroplasty during follow-up (Table 6).

**Table 6:** Postoperative safety events according to final-follow-up MPTA group.

Event	MPTA >95° (*n* = 81)	MPTA ≤95° (*n* = 84)	*P* value
Postoperative thrombosis	2 (2.5%)	3 (3.6%)	1.000
Postoperative wound complications	6 (7.4%)	7 (8.3%)	0.827
Postoperative conversion-to-TKA within 5 years	0 (0)	0 (0)	-

Values are presented as *n* (%). Postoperative thrombosis was compared using Fisher's exact test, whereas wound complications were compared using the chi-square test. No *P* value was calculated for conversion to total knee arthroplasty because no events occurred in either group. Conversion-to-TKA data apply only to the 165 knees with at least 5 years of follow-up included in the final analysis. MPTA, medial proximal tibial angle; TKA, total knee arthroplasty.

## 4 Typical cases

Typical Case 1 (MPTA > 95° group): A 60-year-old female patient with osteoarthritis of the medial compartment of the left knee. Upon admission, her MPTA was 85.5°, WOMAC score was 110, Lysholm score was 18, VAS score was 8, and knee ROM was 100°. Postoperatively, the MPTA was corrected to 96.5°. At the final follow-up, her WOMAC score was 42, Lysholm score was 90, VAS score was 0, and knee ROM was 130° (Figure 1).

**Figure 1:**
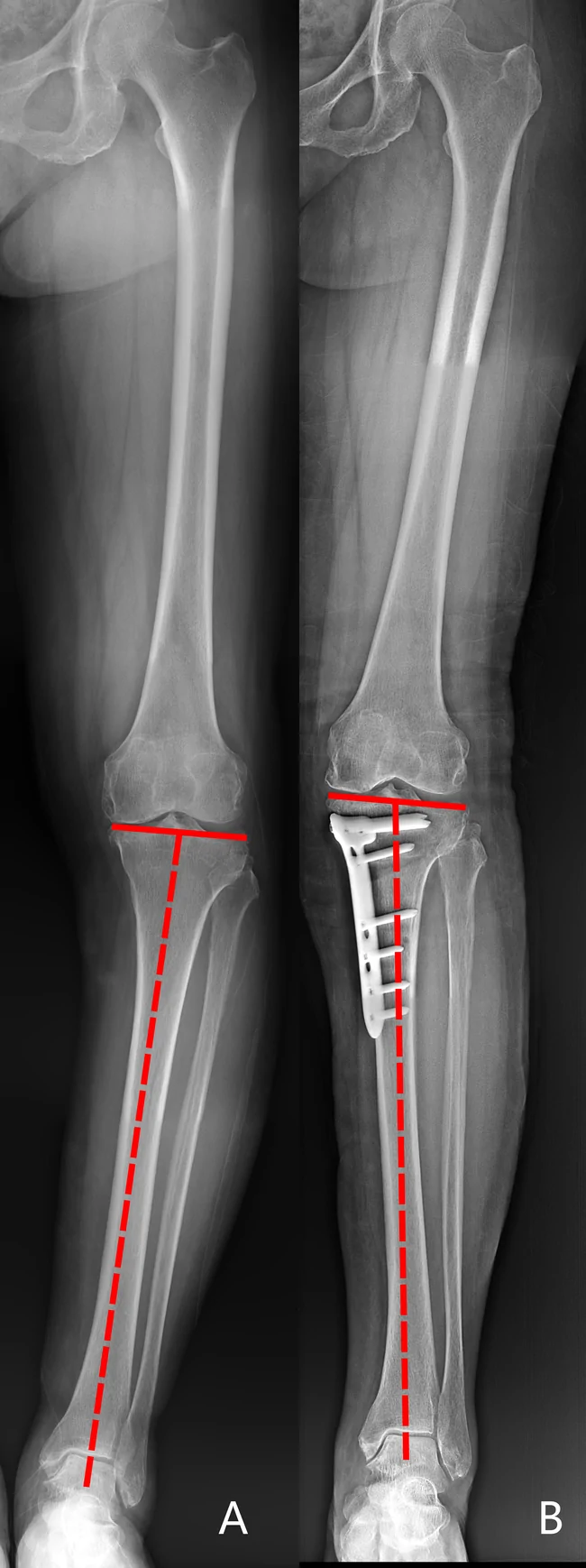
Typical case in the group with a postoperative MPTA > 95° [excessive valgus of the tibia after surgery. **(A)** preoperative, **(B)** postoperative].

Typical Case 2 (MPTA ≤95° group): A 53-year-old male patient with osteoarthritis of the medial compartment of the left knee. Upon admission, his MPTA was 84.3°, WOMAC score was 129, Lysholm score was 21, VAS score was 9, and knee ROM was 95°. Postoperatively, the MPTA was corrected to 91.8°. At the final follow-up, his WOMAC score was 38, Lysholm score was 91, VAS score was 0, and knee ROM was 135° (Figure 2).

**Figure 2:**
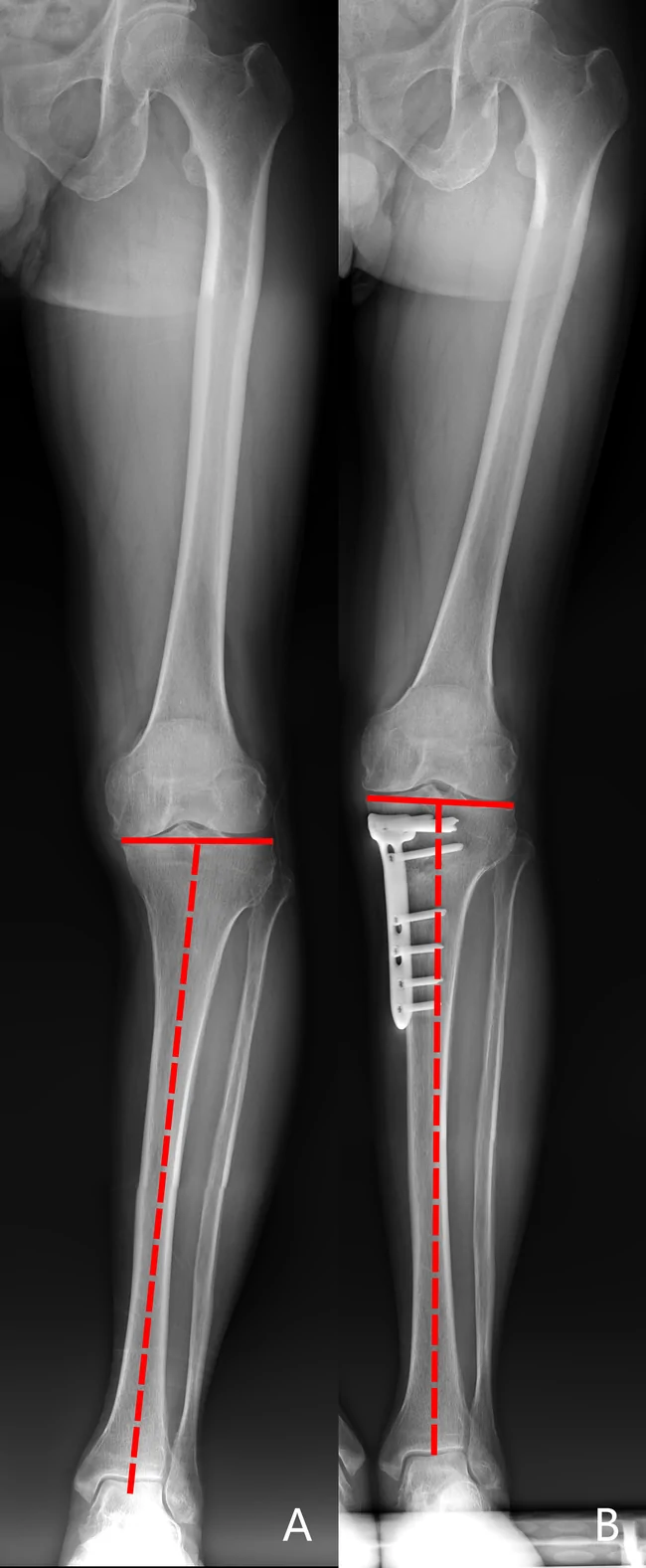
Typical case in the group with a postoperative MPTA ≤ 95°. [**(A)** preoperative, **(B)** postoperative].

## 5 Discussion

### 5.1 Principal findings

The principal finding of this retrospective cohort study was that an MPTA >95° at the final follow-up was not significantly associated with worse mid-term clinical outcomes in patients with K-L grade IV medial compartment knee osteoarthritis after MOWHTO. Overall, WOMAC, Lysholm, VAS, and knee ROM improved substantially from baseline to the final follow-up. Although knees with an MPTA >95° showed greater final MPTA and HKA values and larger changes in coronal alignment, the magnitude of improvement in clinical outcomes was similar between the two groups. After adjustment for the corresponding preoperative values, the between-group differences remained small for WOMAC, Lysholm, VAS, and ROM. These findings suggest that, within this cohort, an MPTA exceeding 95° at approximately 5 years after MOWHTO was not independently associated with poorer patient-reported or functional outcomes. However, the absence of a statistically significant association should not be interpreted as evidence of equivalence or as support for deliberately targeting an MPTA >95° during correction.

### 5.2 Clinical significance of the 95° MPTA threshold

The clinical significance of an MPTA exceeding 95° after MOWHTO remains unsettled. Concern regarding a large MPTA is largely related to excessive joint-line obliquity and the possibility of adverse biomechanical and clinical consequences. Kim et al. reported poorer mid-term radiographic and clinical outcomes when the postoperative MPTA exceeded approximately 95°, supporting the concept of an upper limit for tibial correction (5). Other studies, however, have not reproduced this association. Rosso et al. found that an mMPTA ≥95° was not associated with worse clinical outcomes at a mean 10-year follow-up (7), while another study reported similar overall clinical outcomes at a minimum 4-year follow-up despite greater postoperative MPTA, although lateral compartment pain was more frequent in the increased-MPTA group (6). In a prospective study, Zehir and Alic likewise found no significant relationship between the magnitude of angular correction and postoperative pain or functional outcomes (14). Gimm et al. further reported that postoperative MPTA was not significantly associated with osteoarthritis progression (HR, 1.09; *P* = .135) or conversion to arthroplasty (HR, 1.03; *P* = .755) in the operated knee. Although their endpoints were structural progression and arthroplasty conversion rather than the patient-reported and functional outcomes evaluated in the present study, these findings further suggest that postoperative MPTA alone may not fully determine the prognosis after HTO (15). A recent systematic review concluded that the available evidence remains insufficient to establish a consistent relationship between postoperative joint-line obliquity and clinical outcomes after HTO (4). Our findings add to this literature by focusing specifically on knees with K-L grade IV osteoarthritis, a population in which evidence regarding the clinical relevance of the 95° threshold remains limited.

### 5.3 Radiographic correction and clinical outcomes

Despite the clear differences in final MPTA and HKA between the two groups, the corresponding differences in WOMAC, Lysholm, VAS, and ROM remained small after adjustment for baseline values. This suggests that the mid-term clinical outcome after MOWHTO may not be determined by the magnitude of tibial correction alone. The postoperative mechanical environment of the knee reflects the interaction of osseous alignment, joint-line orientation, intra-articular alignment, and soft-tissue balance. Matache et al. reported that greater lateral knee laxity was associated with an increased risk of excessive joint-line obliquity after MOWHTO, indicating that the effect of tibial correction on the final joint-line orientation may vary according to the soft-tissue characteristics of the knee (16). Kumaki et al. also found that changes in JLCA were associated with postoperative patient-reported outcomes after MOWHTO, supporting the relevance of intra-articular alignment in addition to bony correction (17). These findings may partly explain why a larger MPTA in the present cohort was not accompanied by proportionally worse clinical outcomes. The 95° MPTA threshold should therefore be interpreted within the overall alignment of the knee rather than as an isolated predictor of postoperative function.

### 5.4 Interpretation of JLCA changes

In contrast to the clear changes in MPTA and HKA, JLCA remained largely unchanged in the present cohort, decreasing only from 3.27° preoperatively to 3.21° at the final follow-up. Previous studies have reported more pronounced postoperative changes in JLCA after HTO and have shown that these changes may influence correction accuracy and postoperative outcomes. Na et al. demonstrated that changes in JLCA should be considered when evaluating coronal alignment correction after HTO, while more recent studies have also identified postoperative JLCA change as an important contributor to alignment accuracy (18, 19). The difference observed in our cohort may be related to the characteristics of the study population and the timing of radiographic assessment. All included knees had K-L grade IV osteoarthritis, and JLCA was evaluated on weight-bearing radiographs at the final follow-up rather than during the early postoperative period. JLCA reflects intra-articular alignment and is influenced by the interaction between joint deformity and surrounding soft-tissue laxity rather than by osseous correction alone (20). Its measured value may also vary with loading conditions and knee laxity, which may partly account for differences among studies (16, 21). Therefore, the limited change in JLCA observed in this cohort should not be interpreted as evidence of absent cartilage remodeling or incomplete cartilage recovery. Because the present study did not directly evaluate cartilage status or analyze JLCA as an independent predictor of clinical outcomes, no conclusion regarding cartilage repair or the clinical effect of a relatively large residual JLCA can be drawn.

### 5.5 Clinical improvement in advanced osteoarthritis

Despite the advanced radiographic severity of osteoarthritis in this cohort, substantial improvements were observed in pain, knee function, and ROM after MOWHTO. This finding is consistent with recent evidence suggesting that advanced radiographic osteoarthritis does not preclude meaningful clinical improvement after HTO in appropriately selected patients. A 2024 systematic review reported favorable survivorship and significant improvements in patient-reported outcomes after HTO in patients with advanced medial compartment osteoarthritis (2). Long-term data have also shown satisfactory clinical outcomes and a survival rate of 91.5% at a mean follow-up of 13.6 years after MOWHTO in patients with K-L grade III–IV disease (3). In the present cohort, ROM increased from 98.85° preoperatively to 125.45° at the final follow-up, together with marked improvements in WOMAC, Lysholm, and VAS scores. The magnitude of this improvement, however, should not be attributed to coronal realignment alone. Arthroscopy was performed at the time of MOWHTO, with debridement of unstable cartilage and hypertrophic synovium and treatment of meniscal lesions when indicated, followed by a standardized rehabilitation program. These concomitant procedures and postoperative rehabilitation may have contributed to improvements in pain and motion. Because all knees underwent the combined treatment protocol and no comparison group without concomitant arthroscopy was available, the independent contribution of arthroscopy to the observed clinical improvement cannot be determined.

### 5.6 Limitations

This study has several limitations. First, its retrospective, single-center design is subject to selection bias and residual confounding, and the findings should therefore be interpreted as associations rather than causal effects. Second, although all procedures were performed according to a standardized protocol, three surgeons were involved, and unmeasured differences in surgical technique or perioperative management cannot be completely excluded. Third, the cohort was restricted to knees with K-L grade IV medial compartment osteoarthritis, which limits the generalizability of the findings to patients with less advanced disease. Fourth, radiographic assessment was based on preoperative and final-follow-up images, without immediate or 1-year postoperative measurements. Consequently, the temporal changes in MPTA, HKA, JLCA, and PTSA during follow-up could not be evaluated. Fifth, the numbers of postoperative safety events were small, limiting the statistical power to detect between-group differences and precluding conclusions regarding comparable safety. Sixth, the two groups differed significantly in preoperative MPTA (85.35° vs. 83.95°, *P* = 0.001). Although the covariance models adjusted for the corresponding preoperative outcome, this baseline imbalance may still have introduced residual confounding and may limit the comparability of the two groups. Therefore, postoperative differences in MPTA should be interpreted with caution, as they may partly reflect pre-existing differences rather than the intervention itself. In addition, cartilage status was not directly assessed, and the contribution of concomitant arthroscopic procedures to the observed clinical improvement could not be determined independently. The 15 knees excluded because of insufficient follow-up were not subjected to complete data abstraction; therefore, differences between included and excluded cases could not be formally assessed, and attrition bias cannot be excluded.

## 6 Conclusion

In patients with K-L grade IV medial compartment knee osteoarthritis treated with MOWHTO, an MPTA >95° at the final follow-up was not significantly associated with worse 5-year clinical outcomes, including WOMAC, Lysholm, VAS, and knee ROM. These findings suggest that the clinical significance of the 95° MPTA threshold should be interpreted in the context of overall knee alignment rather than as an isolated determinant of postoperative outcome. Further prospective studies with serial radiographic assessment are needed to clarify the optimal correction range and its long-term clinical implications.

## Data Availability

The raw data supporting the conclusions of this article will be made available by the authors, without undue reservation.
